# Supplementary material for: Content-rich biological network constructed by mining PubMed abstracts
Source: BMC Bioinformatics. 2004 Oct 8;5:147. doi: 10.1186/1471-2105-5-147 (PMC528731; doi:10.1186/1471-2105-5-147)
Supplement: Additional File 2 — The original results of the above study (non-essential files are deleted to keep the file size under the limit set by BMC bioinformatics). [file 1471-2105-5-147-S2.bz2 › chilibotAdditionalFile2/dip50/127ID8609467E471/html/P_N.html]

 


 **P** and **N** 
  
Found 149679 abstracts in PubMed, retrieved 50.  
 

 What does Google say? 
 PDF only 
| .edu only 

---

**Interactive relationship** (e.g. stimulation, inhibition, etc)

**Neutral relationship**- Increases in the proportion of saturated FA and in monounsaturated oleic acid 18 1n 9 and a decrease in the proportion of PUFA of the  **n**  6 and  **n**  3 series were produced in the animals fed a low protein diet  **P**  <.  Ref: 12880108 Lipids, 2003
- The average age of the ST group  **n**  = 10 was 12.2 3.9 years compared to 9.5 3.5 years for the SU group  **n**  = 12  **p**  &gt.  Ref: 12881774 Pediatr CardiolPediatr Cardiol, 2003
- In the eyes that received implant C  **n**  = 6, the mean thickness of the recovered lesions  **n**  = 21 measured 39 24 micro m, compared to 65 30 micro m  **p**  < ; 0.001 for the lesions  **n**  = 39 recovered from the fellow control eyes.  Ref: 12881350 Br J OphthalmolBr J Ophthalmol, 2003
- In the eyes that received implant B  **n**  = 6, the mean thickness of the recovered lesions  **n**  = 31 measured 28 15 micro m, compared to 50 29 micro m  **p**  < ; 0.001 for the lesions  **n**  = 19 recovered from the fellow control eyes.  Ref: 12881350 Br J OphthalmolBr J Ophthalmol, 2003
- In the eyes that received implant A  **n**  = 8, the mean thickness of the recovered lesions  **n**  = 36 measured 32 SD 22 micro m, compared to 52 30 micro m  **p**  < ; 0.005 for the recovered lesions  **n**  = 40 from the fellow control eyes.  Ref: 12881350 Br J OphthalmolBr J Ophthalmol, 2003

**Non-interactive relationship** (e.g. studied together, co-existance, homology, etc.)

- 79% 15 19, of Class I, 86% 12 14 of Class II, and 100% 6 6 of Class III, but none of Class IV patients  **n**  = 8  **p**  <.  Ref: 12880118 J Pediatr Endocrinol Metab, 2003
- In our system, named SCINEX  **P**  screening for interactions between extracellular proteins, proteins of interest were fused to truncated Ire1p so as to substitute its  **N**  terminal lumenal domain NLD.  Ref: 12880949 Biochim Biophys Acta, 2003
- Significant changes were observed in the UV difference absorption spectra and CD spectra of both KWGK and 21 mer duplex upon complex formation even at a very low peptide to nucleotide  **P**   **N**  ratios.  Ref: 12880944 Biochim Biophys Acta, 2003
- These studies have also helped to identify a new chemical transformation of the peptide backbone in the Maillard reaction that lead to beta scission of  **N**  terminal tyrosine side chain and  **p**  hydroxybenzaldehyde formation under both aqueous and nonaqueous conditions.  Ref: 12879488 Biopolymers, 2003
- ... the relationship between SCD activity and the proportion of 18 1  **n**  9 was not significant r2 = 0.552,  **P**  > 0.05.  Ref: 12880109 Lipids, 2003
- A significant correlation was observed between PCE activity and the hepatic proportion of 18 1  **n**  9 r2 = 0.874,  **P**  <.  Ref: 12880109 Lipids, 2003
- Crystal structures of  **N** ,  **N**  m tolyl thiourea and  **N** ,  **N**   **p**  tolyl thiourea.  Ref: 12880100 Anal Sci, 2003
